# Obstacles and opportunities to using research evidence in local public health decision-making in England

**DOI:** 10.1186/s12961-019-0446-x

**Published:** 2019-06-28

**Authors:** Dylan Kneale, Antonio Rojas-García, James Thomas

**Affiliations:** 1 Evidence for Policy and Practice Information and Coordinating Centre, UCL Institute of Education, University College London, 20 Bedford Way, London, WC1H 0AL United Kingdom; 2 NIHR CLAHRC North Thames, Department of Applied Health Research, University College London, 1-19 Torrington Place, London, WC1E 7HB United Kingdom

## Abstract

**Background:**

Local public health service delivery and policy-setting in England was overhauled in 2013, with local government now responsible for the complex tasks involved in protecting and improving population health and addressing health inequalities. Since 2013, public health funding per person has declined, adding to the challenge of public health decision-making. In a climate of austerity, research evidence could help to guide the more effective use of resources, although there are concerns that the reorganisation of public health decision-making structures has disrupted traditional evidence use patterns. This study aimed to explore local public health evidence use and needs in this new decision-making climate.

**Methods:**

Semi-structured interviews with Public Health Practitioners across three Local Authorities were conducted, with sites purposefully selected to represent urban, suburban and county Local Authorities, and to reflect a range of public health issues that might be encountered. A topic guide was developed that allowed participants to reflect on their experience and involvement in providing evidence for, or making a decision around, commissioning a public health service. Data were transcribed and template analysis was employed to understand the findings, which involved developing a coding template based on an initial transcript and applying this to subsequent transcripts.

**Results:**

Increased political involvement in local public health decision-making, while welcomed by some participants as a form of democratising public health, has influenced evidence preferences in a number of ways. Political and individual ideologies of locally elected officials meant that certain forms of evidence could be overlooked in favour of evidence that corresponded to decision-makers’ preferences. Political involvement at the local level has increased the appetite for local knowledge and evidence. Research evidence needs to demonstrate its local salience if it is to contribute to decision-making alongside competing sources, particularly anecdotal information.

**Conclusion:**

To better meet decision-making needs of politicians and practitioners, a shift in the scope of public health evidence is required. At a systematic review level, this could involve moving away from producing evidence that reflects broad global generalisations about narrow and simple questions, and instead towards producing forms of evidence that have local applicability and can support complex policy-focussed decisions.

## Background

Public health practitioners (PHPs) (the core public health workforce) face a remit of significant magnitude, with responsibility for promoting and protecting health and wellbeing, tackling health inequalities, and improving healthcare quality. Within England, alongside these broad responsibilities, PHPs have also recently negotiated the transition of public health decision-making away from the National Health Service (NHS) into Local Authorities (LAs, local government) and Health and Wellbeing Boards, following the implementation of the Health and Social Care Act 2013 [1] (see [2] for a fuller overview of changes). In this new environment, PHPs have faced the challenge of changing hearts and minds in promoting the status of public health within LAs, including among elected councillors, who now hold ultimate decision-making authority in local public health priority-setting and commissioning [3]. More recently, the role of PHPs has been further complicated by a sustained decrease in public health funding, described in a recent King’s Fund blog as “*death by a thousand cuts*” [4]. In real terms, these cuts equate to an over 20% decrease in per person funding for public health in some of the most socioeconomically deprived LAs in England (based on [5]).

Using research evidence judiciously within commissioning and policy-setting offers decision-makers greater access to information on what works, increases the opportunities for the effective use of scarce resources, and improves certainty around the likelihood of success when implementing different intervention options [6]. The use of research evidence within public health decision-making in England has been of concern for a number of public health researchers, as evidenced by the number of studies that have sought to investigate evidence use within new public health decision-making cultures (for example, [7–13]) and systematic reviews that have synthesised evidence on the role of research evidence in decision-making (for example, [2, 14]). Primary studies have variously focussed on the way in which PHPs use evidence within newly created Health and Wellbeing Boards [7, 12, 13], the way in which evidence on social determinants of health is used by decision-makers working outside health [9], or on particular health areas such as alcohol policy [11] or a particular type of evidence such as national evidence-based guidelines [8, 10]. These have reinforced the perception that the reorganisation of public health decision-making has introduced a political dimension to the use of evidence [8, 10, 11, 13], which has disrupted established evidence use preferences [8, 10, 11, 13] and introduced a greater demand for locally sourced evidence [10, 11]. However, our previous systematic review highlighted a need to better understand how disruptions to evidence use patterns in public health have changed decision-making processes, and how evidence generators need to respond [2].

Further understanding the determinants and processes of research evidence use can help to address the challenges of implementing evidence-informed practice [15] in public health. However, to avoid adopting a stance that inherently assumes that underutilisation of evidence in decision-making is due to an evidence gap that can simply be plugged by producing more peer-reviewed research or systematic reviews in the same style [16], we also need to establish how evidence needs to change to better meet the needs of decision-makers. The research presented in this paper builds on our own work [2, 7] and the work of others before us to specifically understand (1) how the reorganisation of public health decision-making from the NHS into LAs has influenced the determinants and processes of evidence use; (2) if there are points in decision-making where evidence could make further contribution; and (3) to understand how changing patterns of decision-making following reorganisation have shaped evidence needs.

## Methods

This study adopts a qualitative approach in order to understand the processes and mechanisms of evidence use in public health decision-making. This paper is based on interviews that were conducted with 12 PHPs situated within public health teams across three LAs. These sites were purposefully selected to represent a diversity of settings in order to reflect a range of public health issues that might be encountered, and were located within and beyond the North Thames area. The three included an urban, suburban and county LA, the latter including large rural areas. The areas also differed in terms of levels of deprivation, with one site falling within the most deprived 20% of LAs in England according to the Index of Multiple Deprivation [17], while the other two fell within the third and fourth quintile. They also varied in terms of political control, with one having been under Labour control (centre-left) for a number of years, another similarly traditionally under Conservative control (centre-right), and a third recently falling under Labour control following a number of cycles of no party having overall control. A broad range of PHPs were eligible, and our sample included Directors of Public Health (1), Deputy Directors of Public Health (2), Public Health Consultants and/or Service Leads and Heads of Service (3), Public Health Intelligence leads (1), Public Health Strategists (1), Public Health commissioning leads (1), Public Health Managers (2), and embedded researchers from Universities (1), although there was overlap in responsibilities and titles across the sites. There was a diversity of experiences among participants with some having worked within LA public health roles previous to the transition of public health responsibilities to LAs, some having joined from NHS roles, and some have joined the public health team from roles external to LAs and the NHS. Furthermore, there was an approximately equal balance of women (*n* = 7) and men (*n* = 5) among the participants. Ethical approval was granted from the UCL Institute of Education’s Research Ethics Committee (REC 862/961).

Interviews were conducted face-to-face and over the telephone between January and August 2017, and lasted between approximately 40 min and 1.5 h. Data collection coincided with political instability at the national level, following just a year after the Brexit vote and during an unanticipated general election held in June 2017; local elections occurred in one of the LAs in May 2017, which saw no change in the overall political control. Interviews were carried out using a mixture of open pre-determined and follow-up questions. The interviews allowed participants to talk about their role and the types of duties that they undertook, their experience of the transition of public health from NHS to LA, and their experience and involvement in providing evidence for, or making a decision around, commissioning a public health service or making a policy change. We also asked participants to reflect on findings from previous research undertaken by the team [2, 7], including on the use of qualitative research, the involvement of local politicians in public health decision-making and the impact on evidence use patterns and requirements, the use of evidence within local public health strategies, and the challenges in identifying relevant evidence for decision-making.

All interviews were transcribed in full and thematised using NVivo 11. We used template analysis as a way of understanding the findings, which involved developing a coding template based on an initial transcript and applied this to subsequent transcripts. This form of analysis of qualitative data is said to strike a balance between imposing a relatively high degree of structure in the process of analysing textual data, but also maintaining flexibility allowing for the emergence of new themes that may be particular to sub-groups of cases. This approach was particularly beneficial in this study as it allowed us to recognise that our role as evidence generators may influence our initial thematising of the data, but the flexibility of the approach also allowed us to develop new concepts that were independent of our roles and discoverable through the research process [18]. The coding template, which involved creating nodes and sub-nodes within NVivo 11, was then revised and reapplied with additional transcripts [19]. The flexibility of this approach differs from other similar approaches, such as framework or matrix analysis, in that themes are drawn directly from the data, rather than from pre-constructed themes drawn from theory or practice [19]. Codes were applied as nodes, both hierarchically and in parallel to the transcripts, and eventually nodes and sub-nodes were developed into themes; these were then reviewed for saliency and richness across cases, although some themes exhibiting stronger in-case or small group significance were also identified and described in the results [19–21].

## Results

Results were thematised into three broad groups, namely evidence-use drivers that reflect the changing landscape of public health decision-making since 2013, evidence-use drivers that we described as ‘entrenched’ that likely pre-date 2013, and unmet evidence requirements that follow from these drivers. These trends take place against a background of cuts to public health budgets among LAs.

### New drivers of evidence use practice: the role of locally elected politicians

Participants spoke freely and sometimes vehemently about how the change in decision-making structures had impacted upon decision-making processes. For a small number of respondents, the transfer of public health commissioning and policy-setting into LA, and the consequent involvement of local politicians in decision-making, brought benefits to public health decision-making, both hypothetical and realised. The politicisation emphasised in earlier studies [13] was viewed as democratisation by one participant, and the new structures were perceived as increasing pragmatism and dynamism to decision-making.

I: *Oh yes. So have you yourself experienced increased political involvement in your work?*

R: *For me it’s called the democratization* [of public health] *(laughs). The other thing to bring to bear is about ways of working and there is a push here, for instance, to be much more action focused and much more agile…. just get it done.*

Other benefits were also described by participants around the increased scrutiny that local politicians could bring to bear on the evidence underpinning large commissioning decisions. This was expressed in terms of the alternative skills, experience and views that decision-makers with no formal public health training could bring to bear on assembling and interpreting evidence. However, there was greater consistency across the majority of participants that a heightened political climate in public health decision-making had, or potentially could, interrupt the flows of evidence to influence policy and practice in three key ways, namely (1) through imposing an ideology to local public health commissioning and policy-setting that was not always commensurate with need; (2) through necessitating evidence to be ‘packaged’ to meet the needs of local decision-makers; and (3) through changing the type of evidence used in order to provide a persuasive case for action.

#### Political ideology and decision-making

The political orientation of a LA was described as influential in the types of services that were commissioned. For example, a more libertarian political orientation was implicitly viewed as being associated with a more medicalised model of public health. This discouraged the commissioning of services that could be viewed as ‘mollycoddling’ or ‘lecturing’ people, as explained by one participant who attributed the discontinuation of smoking cessation services in a neighbouring LA as directly linked to it being under Conservative (centre-right) control.

In one site, the impact of political ideology on public health decision-making was generally described as moderate, although it was recognised that local politicisation of public health could be problematic in other areas.

“*I think that in this context in* [Local Authority] *what we’re trying to achieve as a public health team is fairly strongly in line with the political views of the councillors that we work with…so I haven’t encountered a situation in which it hasn’t been politically convenient to commission something that we have been pushing on based on the evidence.… But it’s really clear that it can and it does happen in other places as well.*”

In a second study site, participants described the way in which the narrow sociodemographic profile of local elected officials could necessitate constructing the case for a service in a different way. For example, as described below, the terms of the argument for providing sexual health services changed from protecting and promoting the psychological and physical wellbeing of the population, to an economic argument around the consequences of ‘unchecked’ sexuality and fertility. In another instance, changing the terms of the debate to a more ‘business focussed’ argument was described as being in conflict with the “*reflective kind of space*” that public health has traditionally occupied.

“*Yes the councillors of course, they all come from different backgrounds, but they are usually a lot older and they like their libraries and they like the fire service and then you’ve got to talk about sex with them and that’s very difficult. But this is where we rely heavily on our director who has worked fastidiously on getting this message across which is: it* [sexual health/ill-health] *does exist, and you do need to know about it, and this is the reason why. And actually* [what] *we can put with that - which always gets the ears open - is the cost associated with the problem…*

*…the other one we use is if you don’t spend this pound* [£] *on contraceptive services it’s going to cost you members of the council through housing and education you know….so I know you don’t want to talk about sex but actually involves really simple things and really cheap things and will cost a fortune if we don’t do it.*”

In a final study site, two participants described, the negative impacts that electoral accountability had in terms of the focus of public health issues. This was not related to ideology with respect to Labour or Conservative allegiance, but more broadly the ideology of being ‘political’. This extended to claims that public health issues could be deliberately overlooked because of concerns about political reputations and electability.

The electoral cycle and political make-up of the council was also described as changing the way in which public health was conceptualised by some participants. Areas with a stable political make-up were those perceived as being able to take more electorally risky commissioning decisions around upstream interventions, and to be able to more fully employ a socioecological (or salutogenic) model of public health.

#### Translating back and forth between evidence and anecdote

Several participants described instances where local elected officials relied on tacit knowledge and wisdom in guiding their decision-making. Across sites, participants described the way in which local elected officials would hold the face validity of evidence and ideas in esteem, and described how research-based evidence could sometimes play a secondary role.

“*I think sometimes it’s the case of getting them* [decision-makers] *to pay attention to the evidence at all and saying: ‘I know you think that sounds like a good idea but actually if you look at the evidence it won’t work because so and so’. I think sometimes there is that thing in actually convincing them to look at the evidence in the first place or even to think about evidence when something might look like a good idea on face value.*”

As is common in previous studies, participants described instances locally elected officials exhibited preferences for anecdotal evidence.

“*I: So which types of evidence have you found are more attractive to politicians?*

*Anecdote! (laughs). ‘Oh I saw this that and the other in my constituency’ that kind of thing.*”

However, local politicians’ preferences for anecdotal evidence was also viewed sympathetically by several participants, who viewed utilising knowledge gained directly from engaging with the electorate as important to their roles.

#### Packaging evidence for lay audiences

Most participants discussed ways in which they presented evidence differently for decision-makers who lacked public health training.

“*I: That’s interesting, so what do you mean by presenting it* [research] *in an appropriate way?*

*R: I mean just because most of those members will not have a public health background and that they …their expertise generally lies elsewhere. Some of them come from a health and social care background and so on, and so will have more of an understanding of how things work within a public health system, but others…. So yeah that’s what I was thinking. Producing reports and things that are jargon free on what needs to be done from the research evidence without going into unnecessary detail and so on. So I think that has to be considered.*”

Some participants described that they had become more ‘politically’ aware in their roles than was previously the case, and had changed the way in which they worked to include a greater focus on developing relationships with decision-makers before and during the assembly of evidence in order to better guarantee that the messages did inform commissioning and policy-setting.

“*But we need to develop relationships with the local cabinet member who has responsibilities for various areas that we want to influence, and that’s much more of something that we have had to learn over time. So, what we do is that… we do briefings to our cabinet members on certain topics and to ensure that they are up to date, they know what the state of play is, they know what the evidence is and… are kind of on board with our way of thinking.*”

Two instances were described where the increased political involvement did change the type of evidence that was drawn upon. Firstly, most participants discussed the persuasiveness of economic evidence in building up a case for commissioning a service or policy among local decision-makers. Economic evidence, and particularly return on investment evidence, fulfilled other properties also highly regarded by participants, including certainty and interpretability, and was viewed as being suited in communicating complex messages.

“*I think people like those. I think there’s not enough maybe. So, people love those infographics – to spend £x on gives £x – in public health we need more. But in public health I think it’s harder...*”

Despite greater demand and usage of economic evidence, and its value in conveying complex messages, participants occasionally described its weaknesses. In some cases, critiques were levelled at the robustness of the underlying assumptions and data when LA sought to generate their own economic evidence. Alternatively, the available economic evidence was critiqued for providing narrow conceptualisations of ‘return’ and ‘investment’ that did not reflect LAs’ broad remit across different services. In addition, economic return on investment evidence was described as showing the value of short-term investments and the resulting long-term savings, which was contrasted with participants’ experience of LAs’ requirement of finding short-term savings on long-term investments.

“*The cabinet meeting which I attended one of the things that was raised was that we had evidence on the impact of obesity on cost to the NHS and also on health impact and so on but we didn’t have evidence on the impact of obesity on social care and we didn’t have evidence of impact on things that the council directly pays for and that was….well it was still approved…. but it would have made our case stronger if we were able to talk about return on investment and related it to what the council pays for.*”

Secondly, qualitative research appeared to occupy an uncertain place and was not uniformly viewed as integral to public health decision-making, perceived by some as an adjunct to enrich or enliven other forms of evidence. One participant suggested that qualitative evidence was deliberately omitted from briefings prepared for locally elected decision-makers because of perceptions that decision-makers would conflate qualitative research with anecdote, the consequences of which could see decision-makers substitute more robust qualitative evidence with their own anecdote, which in turn may be biased or prejudiced. Despite some reservations around how qualitative research should be ‘packaged’ for decision-makers, most participants who discussed qualitative evidence were able to cite examples where this form of evidence had made a valuable contribution in decision-making in uncovering information about hard-to-reach groups or in providing evidence that was otherwise unobtainable about why services had thrived or faltered.

### Entrenched evidence use challenges and practices

#### Highly structured evidence use procedures

Participants described how the use of evidence was inextricably tied to other processes occurring within public health departments that were structured and rigid. Commissioning services was described as a continual and central function of public health departments, and regardless of their actual role, all participants were familiar with the structured processes involved. This meant that gathering evidence to support decision-making, specifically with reference to commissioning, was viewed by some as a contained process, regardless of ambiguities or uncertainties in the evidence.

“*We take a project management style approach. We set out a timeline and we give ourselves six months to do evidence and then we write out the spec*. [specification].”

One of the consequences of a structured approach was that evidence tended to be frontloaded, with little flexibility to incorporate additional evidence after the initial service specification. Potential service providers were required to offer evidence of their capacity and experience in providing a service, although there was no expectation that potential providers would make available published research evidence to support a particular course of action or interpretation of a service specification.

#### Evidence as a specialism

Evidence was viewed as a specialism of public health professionals, and of little interest to the public or other stakeholders. For this reason, the provenance of evidence was omitted in public-facing documents. Nevertheless, public involvement in decision-making was viewed as important, although in one description, the involvement of local people in decision-making was directly viewed as competing (and not complementary) with research evidence in influencing decision-making.

“*R1: So, when I think about the sort of style of our HWS* [Health and Wellbeing Strategy] *- the kind of style of writing - we don’t really cite… there is almost really nothing cited or referenced in that… and part of that is because… the kind of Joe Bloggs is not going to follow up on that sources and references. Actually, what they are interested in is … what the priorities are, what … how that is going to influence the service provision, that kind of thing…*

*R2: …because if you produce research* [it] *doesn’t mean that decisions are made based on what you found. I think that, in fairness, we have to use that situation where you know that decision-making is always going to be local, and sometimes use of* [people’s] *voice tends to trump* [research evidence].”

#### Lack of time and timeliness of evidence

Time pressures prevented some participants from engaging with research evidence to the degree that they wanted, both in terms of a lack of time and the timeliness of evidence. Rigid commissioning processes placed added emphasis on the timeliness of evidence.

“*There is quite a lot of evidence out there so sometimes it’s about how it’s communicated and how you get access to the evidence and you’d look at how it applies. Sometimes it’s about how it fits with your schedule and timing. So we’ve got a timetable for our commissioning and within that I’m looking at the overall commissioning programme for the public health team but within that there are service specialists who are going on training, keeping up to date, and making themselves aware of evidence.*”

A lack of timely evidence also meant that participants would occasionally develop their own solutions to sourcing evidence that better met their timescales.

“*I wish we had up to date data there but the ONS* [Office for National Statistics] *data only comes out every three years….What we’ve done here is we’ve started groups at the* [local] *level simply because we have such a lag in data, so we actually need to know what is going on at this moment in time, so that’s what we’ve actually done* [here].”

### New evidence requirements

#### Demand for ‘Local’ evidence

Participants had a strong affiliation with their LA and maintained a strong organisational identity during interviews. This identity was constructed on the basis of differences from other LAs, even neighbouring areas, and was developed on the basis of differences in the local population demographics, and particularly ethnicity, as well as on the basis of particular facets of organisational culture. In contrast, few participants spoke of public health challenges or a distinctive epidemiological profile as a distinguishing feature of a LA.

“*…And to be perfectly honest between ourselves and* [lists a total of seven contiguous and non-contiguous authorities] *– we’re very different. We have very different populations.*”

Having evidence with local provenance was viewed as a key way of ensuring that evidence influenced decision-making within a LA. This was commensurate with locally elected decision-makers’ preferences, but was also tied to participants’ own conceptualisations of what made their LA unique.

“*The big thing about the* [named Local Authority’s] *way of doing things is that everyone is really driven by actions and seeing impactful things change. I think that kind of evidence locally making an impact is much more powerful than having a NICE* [National Institute of Health and Care Excellence] *strategy. That’s what will get the attention of the CEO* [Chief Executive Officer]*, the mayor and the lead member.*”

Within both urban sites, participants described factors relating to ethnicity and economic status as determinants of whether evidence was salient.

“*Looking at the population and thinking would that work here and how could that work here – those sorts of things. Definitely the demographics – I’d look at the ethnicity but more the economic situation of what was happening with the people – how busy they are. Because a lot of the public health study does rely on the people coming to us. The prevention side in particular is attracting people before an issue gets too large.*”

Within the more rural area there was greater emphasis on understanding differences within the LA between larger county towns and cities, and smaller pockets of rural public health challenges.

Although the high value placed on local evidence may have stemmed from both knowledge of what types of evidence were influential with decision-makers, as well as participants’ own organisational identity, concerns about the generalisability of evidence were also driven by participants’ experience of where national understandings of public health failed to translate to a local level. The example below details one participant’s experience related to a specific policy around wellbeing [22], and the lack of applicability to a defined local population.

“*But it’s interesting because a lot of people are going out doing initiatives based on the five ways to mental well-being*^*1*^*. When we did some research in* [city] *amongst the youngest single mothers they took one look at this at the thing they thought it looked like a bunch of middle-class nonsense. And there’s around in* [city] *in particular…well it’s a very individual place is* [city]*, there’s very low levels of expectation and very low levels of beliefs in self-efficacy so it’s really important to get into the local population and to understand that mind set to see if this thing that’s been tested on a national level actually would work with the people that we wanted it to work with. And the outcome of having done that research is that we might use it as a framework for commissioning but even using the word well-being doesn’t resonate with the local population.*”

^1^Based on a New Economics Foundation Report [22].

#### Increased demand for ‘systems-based’ evidence

Participants reported difficulties in sourcing evidence that reflected the broad remit of the LA and that situated local people and public health issues within broader systems of influence. This included difficulties in obtaining evidence that matched the complex sociodemographic profiles of local people. Participants were also critical of narrow conceptualisation of public health issues within the research evidence, which failed to address broad research questions of interest when setting policy and commissioning services.

“*They* [systematic reviews] *pick up general principles that are self-evident anyway or they are so specific that there is little that is transferable. So a systematic review on dance among women over the age of 75 is quite interesting and potentially quite useful* [but] *that is not that helpful in helping us think how we spend our limited physical activity budget across a number of different options which might be competing for similar resources.*”

One participant also described the focus within evaluation studies on comparing outcomes of intervention participants with usual care or no intervention, and the failure to take into account existing health systems, unhelpful.

“*So it’s not all about having the same population – it’s about what was in place before you introduced this intervention. So here for example, we’ve got a really comprehensive stop smoking service and in* [the next Local Authority] *they don’t. So saying ‘if you reduced smoking by this much this is the impact you’re going to have’…. it just doesn’t mean the same thing because here to reduce smoking by that much it’s going to be really hard and it’s going to be the really hardcore smokers and if you do get it you’re going to get a bigger benefit. In* [the next Local Authority] *it could be people who were going to give up anyway – there aren’t any actual savings.*”

#### Improved relationships with research generators

Participants across sites appeared to value links with research and academic institutions and, when asked, could describe examples of when partnerships had worked well. Mixed fora of evidence generators and users were viewed as a valuable opportunity where both evidence generators and users could collaborate and share knowledge. However, one participant described how such groups were viewed as idealistic and were often started with good intent, only for this effort to fall behind at a later date.

“[These fora] *have then fallen to the wayside sort of thing because it’s not necessarily the highest priority on people’s to do list and the day job gets in the way in the council and then the attendance drops off and then it becomes a bit pointless.*”

One participant described his efforts to improve collaborations with researchers, particularly in terms of obtaining support around the implementation of research evidence, but found that these efforts were often thwarted by a lack of sustained funding.

“*…So that makes it more complex because the research of perspective is arguably over-funded and the implementation aspect is under-funded. So you* [end up] *doing a very robust evaluation of a very under-funded intervention which both researchers and those who have implemented the intervention will be aware of but they wouldn’t be any kind of resource to prevent that.*”

## Discussion

### Summary

The transition of public health from the National Health Service to local government control has had an impact on decision-making and evidence use patterns, although the nature of this is not uniform across LAs and not across PHP accounts.

At its most basic level, the change to local government control has meant that locally elected council members now have ultimate decision-making power, and may be directly involved in policy-setting and commissioning decisions, particularly those involving large budgets. PHPs have responded accordingly in order to ensure that locally elected officials are informed in making decisions. This has included the repackaging of certain forms of evidence into a format that is more acceptable for locally elected officials, in line with broader debates around ensuring that research and evidence is open and accessible to broader public interest and scrutiny [23]. It has also meant that PHPs have started to work in a way that they describe as being more politically aware, but could equally be perceived as developing relational evidence-policy exchanges [24].

PHPs also divulged that the involvement of local politicians in public health decision-making also entailed being mindful of their evidence use styles and preferences. This included engaging local politicians to look beyond the face validity of ideas and instead to engage more closely with the evidence base, responding to preferences for economic evidence, as well as offsetting a tendency for overreliance on anecdote. PHP responses to these demands were often sympathetic, and in many ways these preferences perhaps embody the democratic principles that increased political involvement at a local level would be expected to bring in terms of innovation, scrutiny and accountability in public health decisions and decision-making [25]. For example, a heavier reliance on, or demand for, economic evidence is congruent with the increased budget scrutiny needed in the austere climate in which local politicians are operating. Similarly, drawing on anecdotal evidence could be synonymous with listening to the views of local people; however, this can be problematic when either becomes the sole determinant or source of evidence in decision-making at the expense of the integration of more diverse or rigorous forms of evidence. This study did not find strong evidence that such a scenario took place, although PHPs may place a different slant or emphasis on the evidence that is most persuasive when preparing summaries for local decision-makers. Similarly, there is an inevitability that politics and political ideology will shape how public health is conceptualised locally, and the role of local government in health improvement. Where the intersection of these elements becomes particularly concerning is where the ‘politics’ of electability, or political ideology, overrules an evidence-base suggesting public health action should be taken, or prevents assembly of such a robust evidence base. PHPs suggested that this scenario could take place, with a minority reporting direct experience, although these were not widespread in PHP accounts.

The results of this study point towards local patterns of evidence needs that are unmet from the perspective of PHPs. Political involvement at the local level has reinforced an appetite for local knowledge and evidence. Research evidence needs to demonstrate its local salience if it is to contribute to decision-making alongside competing sources, particularly anecdotal information collected by local decision-makers from their electorate. Habitually, PHPs seek evidence that reflects the smallest possible geographic unit of ‘local’ in an attempt to locate evidence that is useful and implementable, although also describe several evidence requirements that are currently unmet. These unmet needs include evidence that can attest to its local credentials as well as evidence that is ‘mutlifactorial’ in nature and recognises the interdependencies in the way in which public health issues emerge and the actions that need to be taken in response to these. To better meet decision-making needs, a shift in the scope of public health evidence is required, and particularly at a systematic review level, through moving away from producing evidence that reflects broad global generalisations about narrow and simple questions, and rather towards a form of evidence that has local applicability and can support complex policy-focussed decisions.

While this study is based on research conducted in the United Kingdom, and in England specifically, the experiences documented here may be generalisable to other settings where there has been a shift towards devolution of decision-making or a re-organisation of decision-making structures. Many countries have increasingly devolved or partially devolved healthcare systems; for example, in Europe, healthcare systems in Spain, Sweden, Denmark, Norway, and the Netherlands have notably high levels of devolved decision-making [26–28]. Similarly, many European countries have embarked on austerity programmes involving large cuts in public health spending [29]. The analysis presented in this study shows some of the impacts that such reorganisations and cuts to spending have on the underlying processes of public health decision-making. In particular, the mismatch uncovered here, between the evidence needed to help decision-makers tackling broad complex questions in small geographic areas and the evidence generated on narrower questions across broad geographic areas, is likely to resonate across several of the settings described above. In some of these settings, notably Scandinavian countries, devolution of healthcare has long been an integral part of healthcare strategy [27], and it is perhaps significant that some of these countries are well equipped to provide (quantitative) evidence at local levels through central government-funded registers which encompass aspects of clinical healthcare, public health and social care [30].

### Limitations

Limitations to the findings include that these data are based on practitioners’ own perceptions with no verification from other sources (including verification from local politicians) and on a small sample of PHPs working across different roles across three areas. The breadth of the sample in terms of the different PHP roles across three different sites does potentially increase its generalisability in some ways, although it raises the possibility that depth of understanding was compromised for any one public health function and/or setting. The use of template analysis helped to identify when saturation was reached, and although new codes were generated as the template was applied to each new transcript, the number of new codes generated dropped substantially with each successive transcript analysed. Finally, our role as academic researchers enquiring about the use of evidence within public health decision-making could theoretically have influenced some responses [31].

## Conclusions

One of the intentions of moving public health (back) into LAs was that PHPs would be in a better position to influence the social determinants of health across other areas of LA responsibility [32]. We are unable to comment on the success of this ambition, although our research suggests that much of the evidence PHPs routinely encounter may not necessarily be helpful in aiding PHPs to exert this influence. Limitations in much of the evidence base explain why previous studies and reviews have found that evidence from local service evaluations is so highly regarded [2, 10], as it fulfils the properties of local salience and is embedded in the complexity of local systems.

New models of evidence production, which recognise public health interventions and challenges as ‘complex systems’ composed of many different components that interact to produce an outcome, and attempt to identify the actions needed to change the system in favourable way [33], are an opportunity to provide the type of evidence that is useful for public health decision-making. From a systematic review perspective, this involves recognising public health as an outcome of interlinked elements within a connected whole [34], and potentially synthesising diverse data as a means of understanding how complexity impacts on interventions in specific contexts [35]. In addition, new methods of understanding how local contextual factors can influence the delivery and effectiveness of interventions, such as the integration of local data into transferability estimates, need to be tested [36]. These methods of handling complexity and contextual information represent important opportunities to better align evidence generation practice with decision-making needs.

Alongside the shift in perspectives and methods described above, our research also signals that public health researchers should be prepared to, and be funded to, spend much more of their time working with PHPs to understand how their evidence can be implemented in local settings, avoiding the current ‘publish and run’ model of evidence production. Our findings also suggest that, while the efforts of organisations such as Public Health England to provide training and enhance the capacity of PHPs to interpret evidence are welcome [37], some of these efforts need to be spent offering training for locally elected members on public health and how to interpret and critically appraise different forms of evidence.

Research evidence is only one of many sources that contribute to public health decision-making [38], as echoed by PHPs in this current research, and examining and critiquing patterns of evidence use in public health decision-making may therefore appear superfluous, particularly in the austere climate in which PHPs currently operate. However, the evidence-base suggests that implementation of public health interventions can lead to substantial returns on investment, in the region of 4:1 for local interventions [39]. Achieving such returns is in large part dependent on mobilising a research evidence base that provides robust evidence on what works and how. Our findings also suggest that this evidence base needs to better reflect the complexity of local populations and systems of influence in order for this evidence to be more useful and usable in local public health decision-making. While much of the paper may be focussed on some of the obstacles to evidence-informed public health decision-making, many of the evidence needs described represent opportunities. As has been discussed in earlier studies, there is a healthy appetite for using evidence in local decision-making [7], and many of the evidence needs around greater consideration of context and the need to provide evidence on more complex questions correspond with the direction of travel in new methods of evidence generation [34]. However, these approaches also increasingly blur distinctions between research and implementation and, in order to realise these opportunities, we may need to dramatically rethink artificial boundaries between implementation and research.

## Abbreviations

LA: Local Authority
NHS: National Health Service
PHP: Public Health Practitioner
